# Interobserver agreement of computed tomography in detecting calcified intervertebral discs in comparison with radiography in a population of 13 healthy British Dachshund dogs

**DOI:** 10.1002/vro2.59

**Published:** 2023-03-28

**Authors:** Sara Formoso, Sam Khan, Mark Lowrie, Jonathan Hughes, Paul Freeman

**Affiliations:** ^1^ Department of Small Animal Medicine Queen's Veterinary School Hospital University of Cambridge Cambridge Cambridgeshire UK; ^2^ Dovecote Veterinary Hospital Derby Derbyshire UK; ^3^ Davies Veterinary Specialists, ManorFarm Business Park Higham Gobion Hitchin UK

## Abstract

**Background:**

The aims of this retrospective study were to estimate interobserver agreement in detecting disc calcification with computed tomography (CT) and to compare the number of calcified intervertebral discs identified on CT and radiography in healthy British Dachshund dogs that underwent a screening programme. The current screening programme uses radiography to identify calcified intervertebral discs.

**Methods:**

Healthy Dachshunds aged between 2 and 5 years presenting for spinal radiography and CT as part of a disc scoring scheme were included. The spinal radiographs were scored by an independent assessor as per the screening programme protocol. The CT images were blinded and reviewed by three different observers of differing levels of experience. The number of discs identified as being calcified was then compared between imaging modalities and between observers.

**Results:**

Thirteen dogs were included. Overall, 146 calcified discs were identified by CT compared with 42 by radiography. There was an almost perfect agreement among the three observers identifying calcified discs with CT images (*k* = 0.92). There was a significant difference between the radiography scores and CT scores.

**Conclusions:**

This study demonstrated a significant difference in the number of calcified intervertebral discs identified in the vertebral column of a small population of healthy Dachshunds between CT and radiography. Given the high agreement between the observers with CT, this may be a reliable method for assessing disc calcification in Dachshund dogs and could be a good candidate for future breeding schemes.

## INTRODUCTION

Intervertebral disc extrusions (IVDEs) are a common cause of myelopathy in Dachshunds,^1^, ^2^ with an estimated prevalence of between 11.0% and 15.7% in the UK population,^3^, ^4^ with a contribution from both genetic and lifestyle factors. The comparatively high prevalence in Dachshunds, along with other chondrodystrophic breeds, is attributable to chondroid metaplasia which leads to intervertebral disc dehydration and calcification.^1^ This is then thought to predispose the disc to herniation under minimal stress.

While the specific link between calcification and extrusion has not been elucidated, calcification identified on radiography between the ages of 2 and 4 years has been successfully correlated with an increased likelihood of suffering future disc extrusion. Dogs with five or more calcified discs have been estimated to be 18 times more likely to suffer a disc extrusion than those with none.^5^, ^6^, ^7^ Furthermore, a strong heritability (0.15–0.87) of disc calcification has been shown in Dachshunds,^8^, ^9^, ^10^, ^11^, ^12^ which has led to disc calcification forming the basis for various breeding schemes in Europe and the UK.^12^, ^13^, ^14^

Various breeding schemes are in existence in different countries aimed at reducing the incidence of IVDEs in Dachshunds.^12^, ^13^, ^14^ Vertebral column radiographs have been used for scoring the number of calcified discs in different countries and, since November 2016, a scheme has been available in the UK. The age range for most of the screening programmes is 24–48 months, based on the fact that the number of calcified discs seen radiographically seems to reach a steady level between 24 and 27 months of age; the number may reduce after 4 years.^15^ Dogs screened are given a grade from 0 to 3 according to the number of calcified discs identified. Based on the grading, a recommendation about breeding is made. However, breeding two grade 0 dogs cannot guarantee that the offspring will be free of IVDEs.

Despite the demonstrable heritability of calcification and its link to future extrusion, there is no evidence that the effectiveness of the breeding programmes is decreasing the incidence of IVDE in Dachshunds over time. There are many reasons why this may be the case, including the low sensitivity of radiography for detecting visible calcification when compared to histopathology,^16^, ^17^ and apparent scorer subjectivity, especially between differing levels of experience.^18^, ^19^ While the number of calcified discs is still the best predictor of lifetime IVDE,^6^, ^7^ the combination of these factors brings into question the validity and robustness of the use of spinal radiography in scoring schemes.

It has been suggested that computed tomography (CT) could overcome many of the limitations of radiography by reducing anatomical superimposition and providing improved contrast resolution.^20^, ^21^ In fact, CT has been reported to have a sensitivity of 100% for intervertebral disc calcification in Dachshunds undergoing hemilaminectomy due to IVDE when compared to histopathology.^16^ However, one study showed that CT did not have greater repeatability or reproducibility when compared to radiography, although it was not subject to the same scorer subjectivity.^19^ In order to replace radiography in breeding schemes, CT must not only be accurate when compared to histopathology, it should also show greater precision and repeatability across observers.

The aims of this study were therefore to estimate the interobserver agreement in identifying the number of calcified discs on CT across a range of experience levels. To compare the number of calcified intervertebral discs identified on CT and radiography. We hypothesised that significantly higher numbers of calcified discs would be identifiable on CT and that there would be excellent interobserver agreement.

## MATERIALS AND METHODS

Dachshunds registered with the UK Kennel Club, aged 2–5 years, presenting to a single veterinary centre for the Dachshund Breed Council intervertebral disc disease screening programme between September 2017 and September 2019 were retrospectively identified. To be included, dogs were required to be healthy without any underlying or previous neurological disease, and they had undergone both spinal radiography and CT scans.

All dogs were sedated with 0.01 mg/kg medetomidine (Domitor; Vetoquinol, Towcester, UK) and 0.1 mg/kg butorphanol (Torbugesic; Zoetis, Leatherhead, UK) administered by either intravenous or intramuscular routes. Dogs underwent a minimum of four (miniature Dachshunds) or five (standard Dachshunds) right lateral spinal radiographs as per the UK Dachshund breeding scheme. This was to ensure that all intervertebral disc spaces between C2 and S1 were clearly visible using a Nova HF Atlas 30 kW HF x‐ray pedestal unit (Celtic SMR, Haverfordwest, Wales). The system used was computed radiography, no grid was used, the kV was 50–60 and the mAs was up to 6. All dogs also underwent CT of the entire vertebral column in dorsal recumbency using a 16‐slice multidetector CT helical scanner (Toshiba, Aquilion, Canon Medical Systems, Crawley, UK). The CT imaging conditions were standardised through a spinal protocol using the following acquisition parameters: pitch factor of 0.938 and helical pitch of 15.0, thickness of 0.5–1 mm (depending on the weight of the dog), amperage between 30 and 150 mA (depending on the weight of the dog) and tube peak voltage of 100–120 kV. The dogs were scanned in dorsal recumbency. Radiographs were submitted for scoring (counting of the number of visible calcified discs) to a single highly experienced scorer in accordance with the current Dachshund breeding scheme (observer 4). The latter is one of the official scorers of the breeding scheme with great experience reviewing radiographs.

The CT studies were blinded and independently reviewed by three observers. The three observers were a third‐year resident in veterinary diagnostic imaging (observer 1), a diploma holder in veterinary neurology (observer 2) and a first‐year resident in veterinary neurology (observer 3). All observers reviewed the images in Digital Imaging and Communications in Medicine format. The images were reconstructed in high‐frequency algorithms and reviewed in a bone window (window width 2500 and window level 500) in 0.5–2 mm slices. Multiplanar reconstruction was used to review the spine in transverse, sagittal and dorsal planes. Each observer assessed every intervertebral disc from C2 to S1 and scored if they were calcified or not (yes or no) for each disc of each dog. Calcification was defined as a subjective assessment of presence of mineral attenuating material visible within the intervertebral disc. Mineral attenuating material was defined as a substance with attenuation properties similar to those of the adjacent vertebral end plates. The information of each observer was recorded in an Excel table and collated. The mean number of calcified discs identified on CT for each dog was calculated between all observers (Table 1).

**TABLE 1:** Summary of observer findings in 13 dogs with calcified discs identified with radiography and computed tomography

	Calcified discs seen with computed tomography					
Dogs evaluated (*n* = 13)	Observer 1	Observer 2	Observer 3	Mean	Two observers (mean observer 1 and 2)	Calcified discs seens with radiography ‐ Observer 4
1	5	5	8	6	5	2
2	14	13	14	13.66	13.5	3
3	15	17	16	16	16	10
4	3	4	4	3.66	3.5	2
5	14	13	14	13.66	13.5	7
6	10	10	10	10	10	3
7	16	18	18	17.33	17	1
8	14	12	14	13.33	13	0
9	13	13	13	13	13	1
10	12	14	13	13	13	5
11	14	14	14	14	14	2
12	13	12	12	12.3	12.5	6
13	0	0	0	0	0	0
Total	143	145	150	145.94	144	42

### Statistical analysis

Data were introduced and collated in Microsoft Excel 2019 (Microsoft Corporation, Redmond, WA, USA) and all statistical analyses were conducted using R (version 4.0.2; R: a language and environment for statistical computing; R Core Team, R Foundation for Statistical Computing, Vienna, Austria; 22 June 2020; www.R‐project.org). The data and the statistical analysis are available for consultation. The data were tested for normality of distribution using the Shapiro–Wilk test and normal Q–Q plots. Equality of variance was tested using Bartlett's or Levene's test where applicable. For all statistical analyses, *p*‐value less than 0.05 was considered significant.

The interobserver agreement was calculated with weighted, non‐weighted and Fleiss’ kappa for three reviewers who performed CT scoring.^22^ The data were subdivided by region (cervical, thoracic and lumbar) and the analysis was repeated. As suggested,^22^ the strength of agreement beyond chance for different kappa values was poor (less than 0), slight (0–0.20), fair (0.21–0.40), moderate (0.41–0.60), substantial (0.61–0.80) and almost perfect (0.81–1.00). All kappa values were calculated with 95% confidence interval (CI).

Interobserver comparisons of CT and radiography scores were undertaken using ANOVA for normally distributed data and Kruskal–Wallis rank sum test for data that were not normally distributed. Again, the data were subdivided by region and the analysis was repeated. Comparison between all CT observers and the official radiography score was also performed using a Kruskal–Wallis rank sum test and post hoc Dunn test.

## RESULTS

A total of 13 entire Dachshunds were included in the study, six males and seven females. The mean age of the dogs at presentation was 35 months, varying from 24 to 61 months of age. A total of 338 discs were assessed and scored (26 from each dog). A mean of 146 calcified discs were identified with CT (Figure 3) by the three observers while 42 calcified discs were visualised with radiography (Figure 2) by observer 4 (see Table 1).

### Agreement

For the CT images, Fleiss’ kappa demonstrated almost perfect agreement between the three observers (*k* = 0.92, *z* = 29.3, *p* < 0.01, 95% CI). When repeated by region, Fleiss’ kappa also showed almost perfect agreement for the cervical (*k* = 0.95, *z* = 14.5, *p* < 0.01, 95% CI), thoracic (*k* = 0.92, *z* = 20.7, *p* < 0.01, 95% CI) and lumbar regions (*k* = 0.89, *z* = 14.7, *p* < 0.01, 95% CI), with the best agreement being in the cervical region.

### Interobserver and intermodality comparison

**FIGURE 1:**
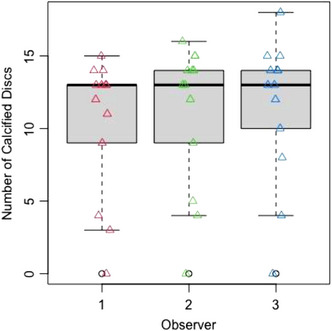
A boxplot of the numbers of calcified discs identified on computed tomography scan images by three observers (median with box showing 25 and 75 percentiles and outlying range).

For CT calcification scores, a one‐way Kruskal–Wallis rank sum test showed that the median number of calcified discs did not differ significantly between reviewers (KW = 1.15, df = 2, *p* = 0.56; see Figure 1).

**FIGURE 2:**
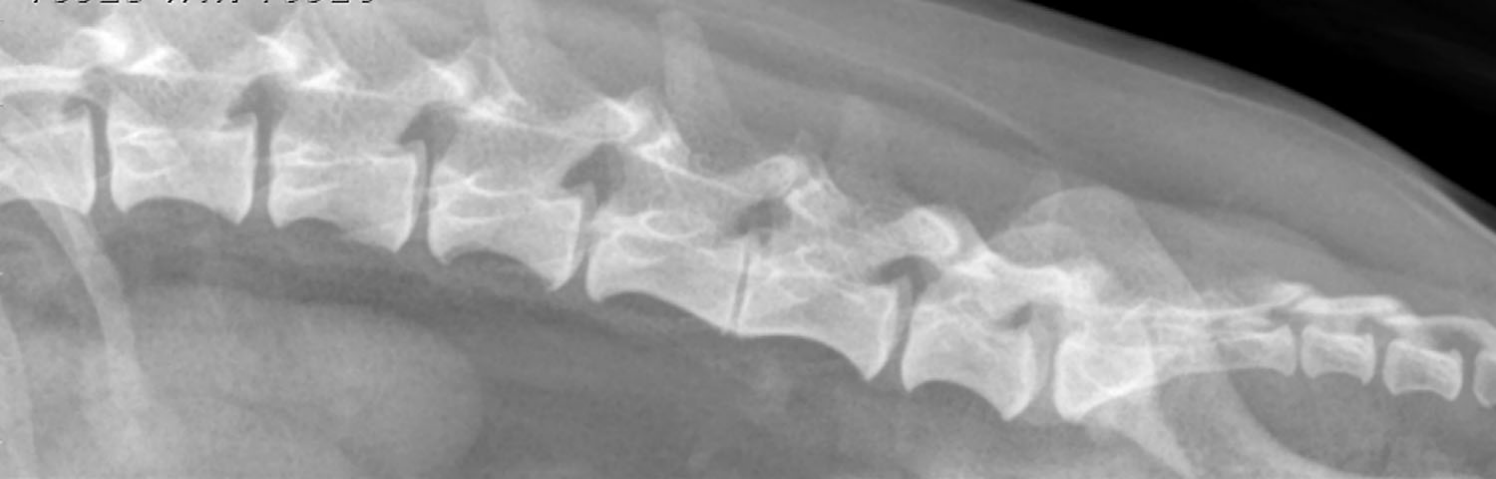
Lateral radiograph of the lumbar spine of a Dachshund. The L1–L2 intervertebral disc space appears calcified.

**FIGURE 3:**
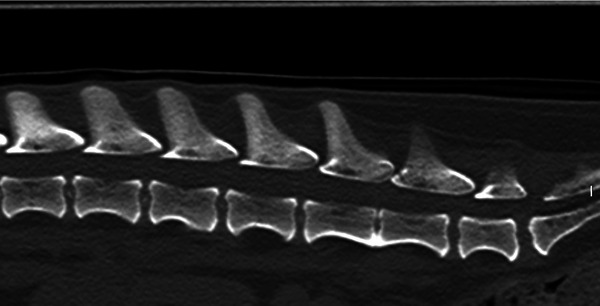
Sagittal reconstruction computed tomography scan of the same area in the same dog in Figure 2 using a bone window. The L1–L2, L2–L3, L3–L4 and L4–L5 intervertebral disc spaces appear calcified.

The data were subdivided by spine region with a one‐way ANOVA showing that the mean number of cervical discs identified as being calcified did not differ significantly between reviewers (*F* = 0.10, df = 2.36, *p* = 0.91), and a Kruskal–Wallis rank sum test showing that the median number of thoracic (KW = 0.53, df = 2, *p* = 0.77) and lumbar (KW = 36,653, df = 2, *p* = 0.83) discs identified as being calcified also did not differ significantly between reviewers.

The scores generated by the three CT reviewers and the official radiography reviewer were correlated using a one‐way Kruskal–Wallis rank sum test, which showed a significant difference in the mean number of calcified discs identified (KW = 17.88, df = 3, *p* < 0.01). A post hoc Dunn test showed a significant difference between the radiography scores and CT scores of all reviewers; furthermore, there were no significant differences between CT scores (see Table 2).

**TABLE 2:** Summary of post hoc Dunn test results for significant differences between the radiography scores and computed tomography (CT) scores for all reviewers

	Observer 4 (radiography)	Observer 1	Observer 2
Observer 1 (CT)	<0.01^a^		
Observer 2 (CT)	<0.01^a^	0.25	
Observer 3 (CT)	<0.01^a^	0.22	0.47

^a^ Significance.

## DISCUSSION

In this study, significantly more calcified intervertebral discs were identified in the vertebral column of healthy Dachshunds using CT than radiography. In total, 146 calcified discs were identified with CT and only 42 were identified on radiography. Furthermore, there was no significant difference in the number of calcified discs identified by each CT reviewer and interobserver agreement was excellent, demonstrating that CT was both precise and reliable.

Our findings were largely in agreement with a previous study,^19^ which reported that CT identified more calcified discs when compared to other modalities (radiography and magnetic resonance imaging) in a population of healthy Finnish Dachshunds. Moreover, our results showed closest agreement among observers identifying calcified discs on CT in the cervical region when compared to thoracic and lumbar regions. A possible explanation may be that intervertebral discs in the cervical region presented with a stage of calcification that was more easily visible with CT; however, without histopathology and mineral analysis, this is speculative and cannot be confirmed.

The level of experience interpreting CT images varied substantially between the three scorers, from a third‐year resident in radiology to a diploma holder in veterinary neurology with more than 30 years of experience reading vertebral column radiographs and a first‐year resident in neurology. In spite of the different levels of training, experience and background, the agreement identifying calcified discs in CT was almost perfect. This may reflect that ability to score with accuracy is independent of level of experience. This is in agreement with a previous study^19^ that reported a lack of individual scorer subjectivity with this modality. Nevertheless, in the present study, the less experienced scorer tended to overinterpret calcified discs suggesting some degree of subjectivity, especially when the focus of mineralisation was small. The authors^19^ also reported a poorer agreement between CT scores when compared to radiographs identifying calcified discs, which may be related to the scorer's experience with the modality.

Difficulties encountered with radiography when assessing possible calcified intervertebral disc spaces are superimposition of anatomy and beam divergence. In contrast, CT offers increased contrast resolution, maintains spatial resolution and allows the possibility to reconstruct the anatomy in multiple planes.

Although our results show that CT scores are repeatable across a range of experience levels, this does not mean that CT can be simply applied to the current Kennel Club scoring scheme. Currently, radiography scores are used to place dogs into categories based upon the risk of developing IVDE and the heritability of calcification, with the most severely affected dogs being advised to be removed from the breeding pool. If our CT scores were applied to the same categories, many more dogs would be removed from the breeding population, potentially creating a genetic bottleneck. It is also not known if CT scores will correlate with risk of future IVDE or if calcification identifiable on CT is heritable. If CT were ever to be used as the method of scoring dogs for breeding purposes, a modified grading system would be needed to account for the significantly higher sensitivity of CT for identifying disc calcifications and to preserve a reasonable breeding pool.

This study had several limitations. Firstly, the sample size was small and derived from a single centre, which makes applying the results to the wider population potentially challenging. Secondly, CT images were only reviewed once by each observer, so no intraobserver agreement could be calculated. The CT reviewers were also all from the same institution and so were likely to be trained in a similar way making it more likely that similar scores would be achieved. In addition, observer 4 scored the radiographs as part of a screening protocol and might have evaluated intervertebral disc calcification more cautiously. That is, mark them as normal when in doubt. It is also possible, however, that the reverse is true and that in order to provide as robust a scheme as possible, this observer marked the discs as calcified when in doubt.

Lastly, identification of disc calcification was made subjectively rather than being based upon specific thresholds of Hounsfield units (HU) and there was no histopathological confirmation of calcification. It is, however, unlikely that the use of specific HU thresholds would have assisted in identification of calcification since herniated discs have been shown to have a wide range of HU (122–913 HU),^23^ and subjective identification of calcification on CT has been demonstrated to have a 100% sensitivity when compared to histopathology.^16^ Future schemes could even rely on a machine learning approach to identification of disc calcification.

In summary, the CT method has the potential to identify a greater number of intervertebral disc calcifications than radiography. Even so, further study is required to elucidate a correlation with future IVDE and heritability before it could be considered for use as the basis for a breeding scheme.

## AUTHOR CONTRIBUTIONS

Paul Freeman conceived of the presented idea contributing to conceptualisation, methodology and writing the original draft. Sam Khan contributed to statistical analysis. Mark Lowrie contributed to collecting data. Jonny Hughes contributed to collecting data. Sara Formoso contributed to collecting data, initial statistical analysis and writing the original draft. All authors discussed the results, contributed to the final manuscript and approved the final version.

## CONFLICTS OF INTEREST STATEMENT

The authors declare they have no conflicts of interest.

## FUNDING INFORMATION

The authors received no specific funding for this work.

## ETHICS STATEMENT

No specific ethical approval was required for this study with all clients being fully informed and giving their signed consent for the details and work to be carried out. The Department of Veterinary Medicine, University of Cambridge Ethics and Welfare Committee approved an application (CR441) for this study to continue in the future so that more cases can be collected if needed for future work.

## Data Availability

Raw data and statistical output are available on reasonable request.
